# Supplementary material for: Prenatal Low Testosterone Levels Induced by DNAH8 Dysfunction Leads to Urethral Fusion and Male Differentiation Abnormalities
Source: Biomedicines. 2025 Dec 10;13(12):3032. doi: 10.3390/biomedicines13123032 (PMC12730954; doi:10.3390/biomedicines13123032)
Supplement: Supplementary file 1 [file biomedicines-13-03032-s001.zip › biomedicines-4005178-supplementary.pdf]

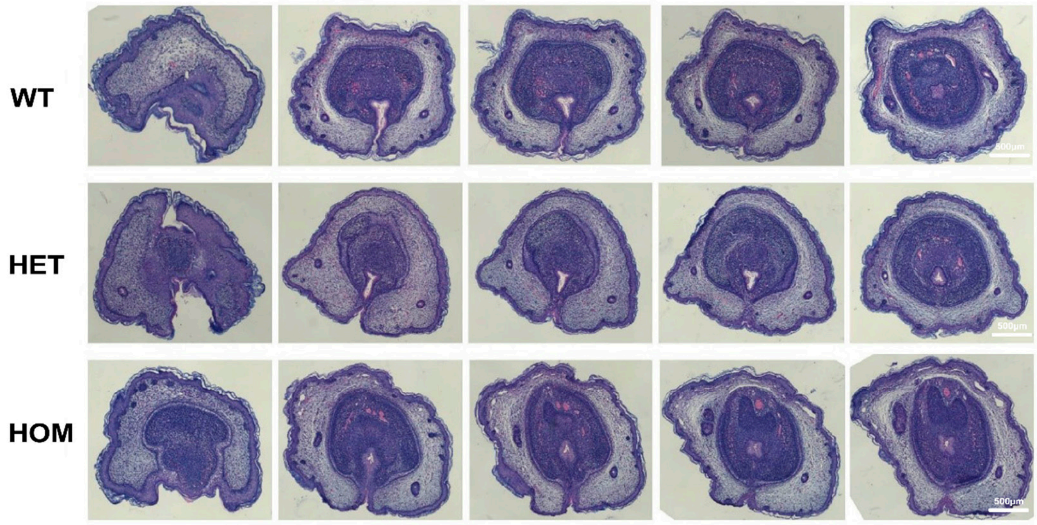

Figure S1. HE staining of the external genitalia of WT, HET, and HOM mice (after birth)

As moves from the base towards the tip of the penis, the cross-sectional area of the penis progressively decreases. In HET staining, no notable differences were detected among the penises of the three groups of mice, and no cases of hypospadias were observed in the HET and HOM groups. As observes the tip of the penis moving towards the base, it becomes evident that the epithelium of the bilateral urethral plates continuously migrates towards the ventral midline, eventually fusing together to create a urethral suture, thereby forming a complete urethral cavity.

Scale bar: 500 μm.

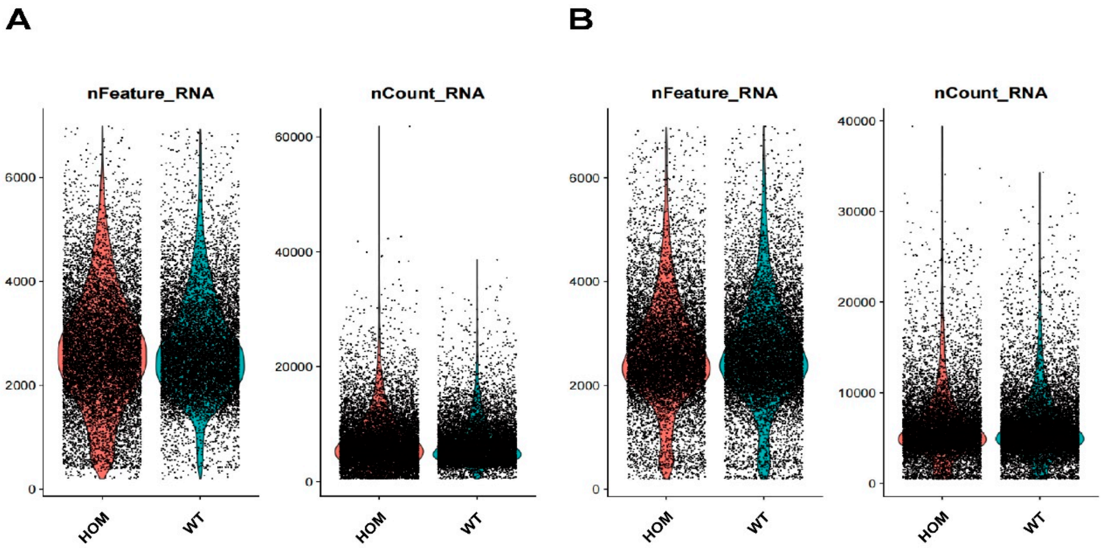

Figure S2. Violin plot showing the total expressed gene count or read count in each sample.

(A) Violin plot showing the total expressed gene count or read count in WT and HOM mice external genitalia samples after quality control and preprocessing.

(B) Violin plot showing the total expressed gene count or read count in WT and HOM mice testis samples after quality control and preprocessing.

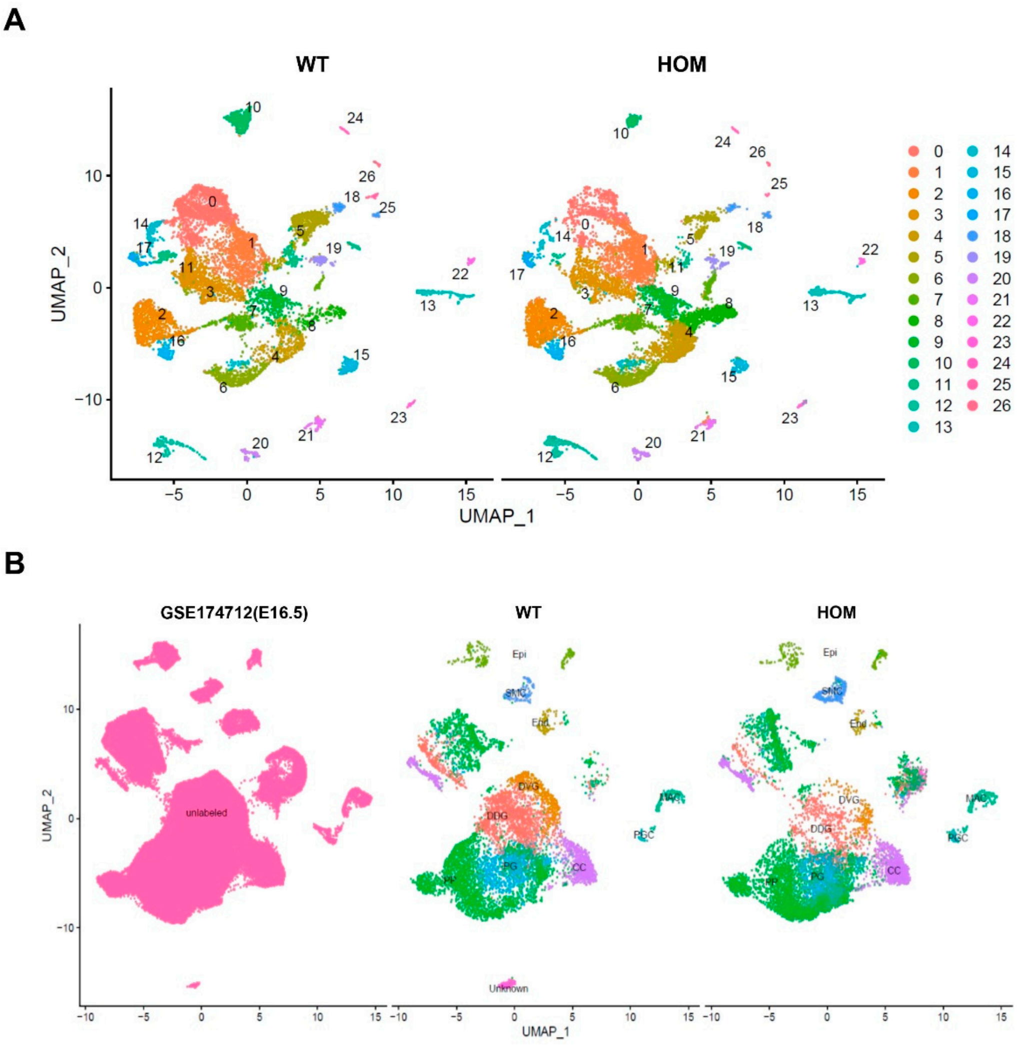

Figure S3. UMAP plot of cells from the external genitalia samples.

(A) UMAP representation of the external genitalia single cells colored by Leiden clustering and 26 cell clusters were obtained.

(B) UAMP dimensionality reduction analysis was performed together with E16.5 mouse external genitalia cells in the GSE174712 single-cell dataset.

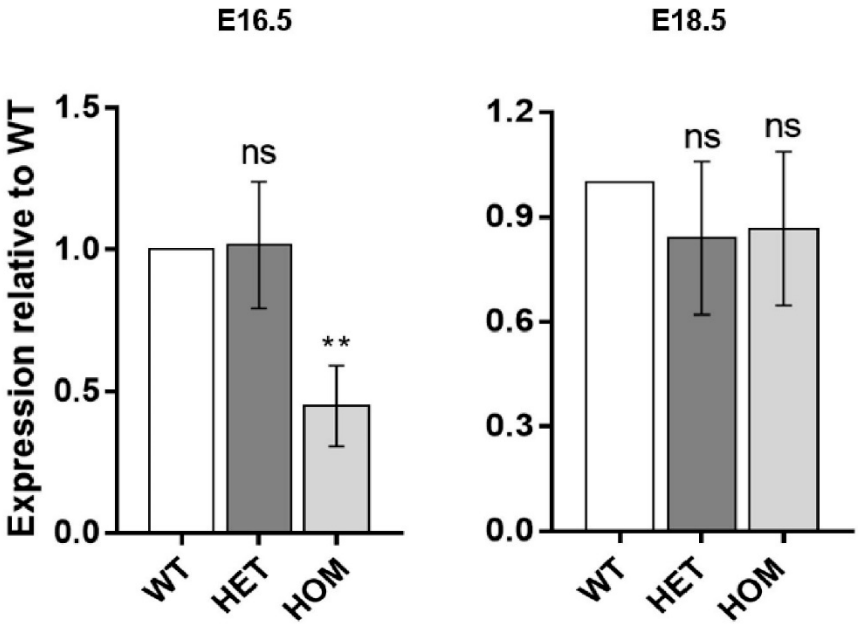

Figure S4. *Ar* expression in mice testes at E16.5 and E18.5.

Error bars are ± s.d. $^{*}P < 0.05$, $^{**}P < 0.01$. ns: no significance.

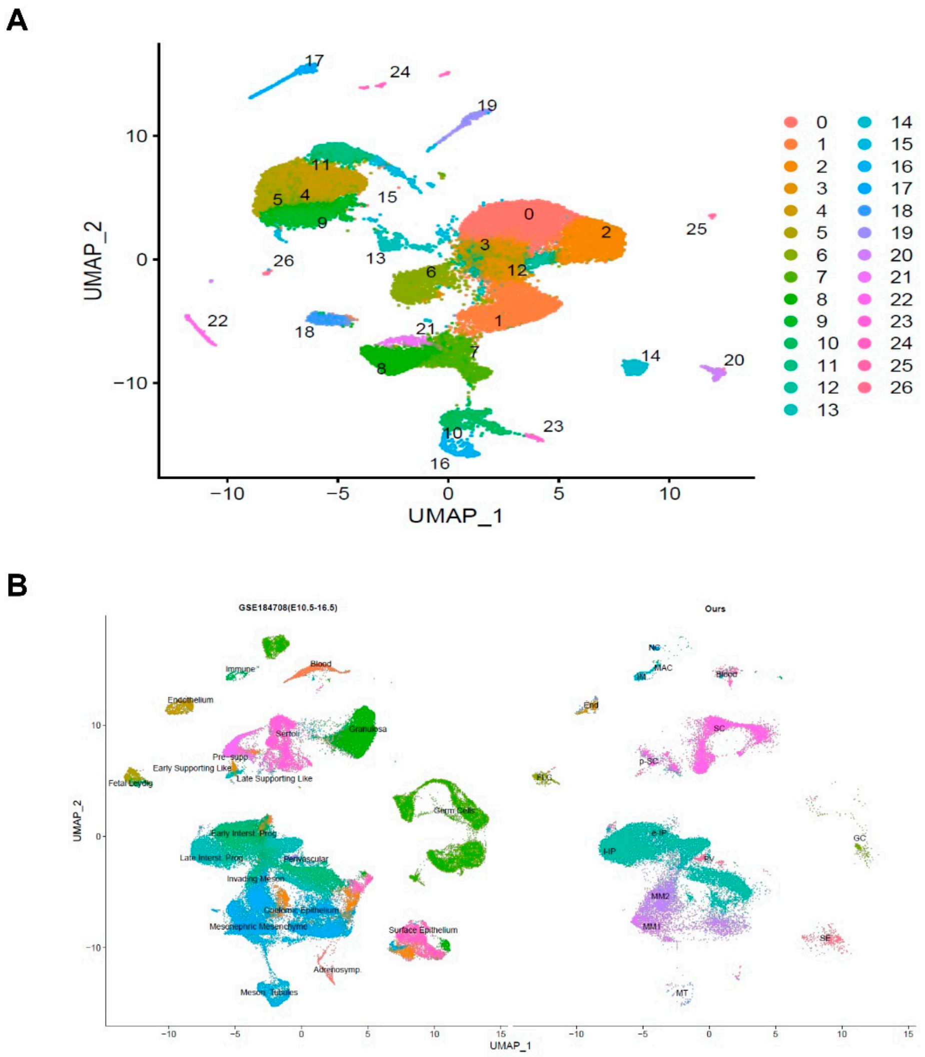

Figure S5. UMAP plot of cells from the testis samples.

(A) UMAP representation of the testis single cells colored by Leiden clustering and 26 cell clusters were obtained.

(B) UAMP dimensionality reduction analysis was performed together with mouse testis cells in the GSE184708 single-cell dataset.

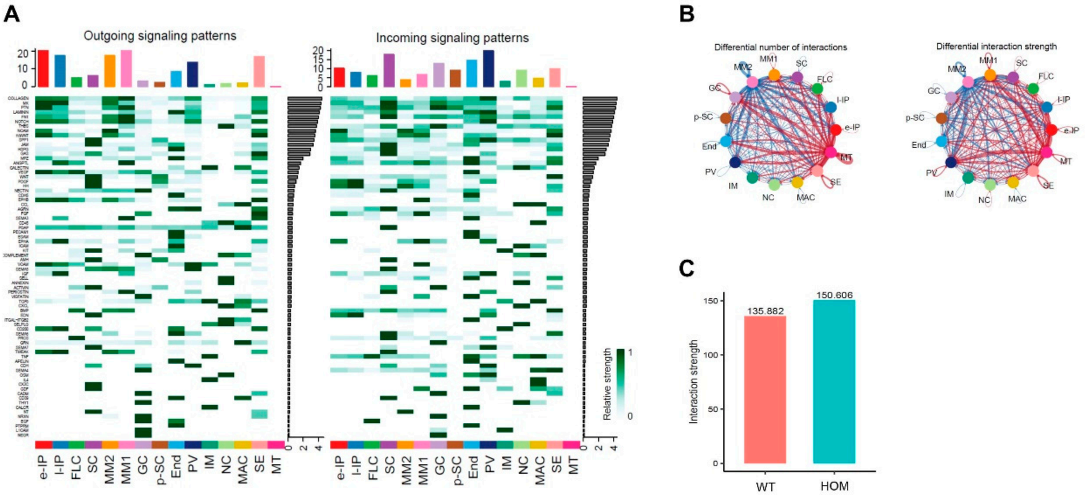

Figure S6. Cell–cell communication network within the testis microenvironment.

(A) Heatmap of the CellChat signalling in each testis subcluster.

(B) Circle plots showing the up- or down-regulation of cell–cell communication network between HOM and WT group.

The red line represents up-regulated interaction and the blue line represents downregulated.

(C) The barplot showing the total interaction strength within testis microenvironment of WT and HOM group.

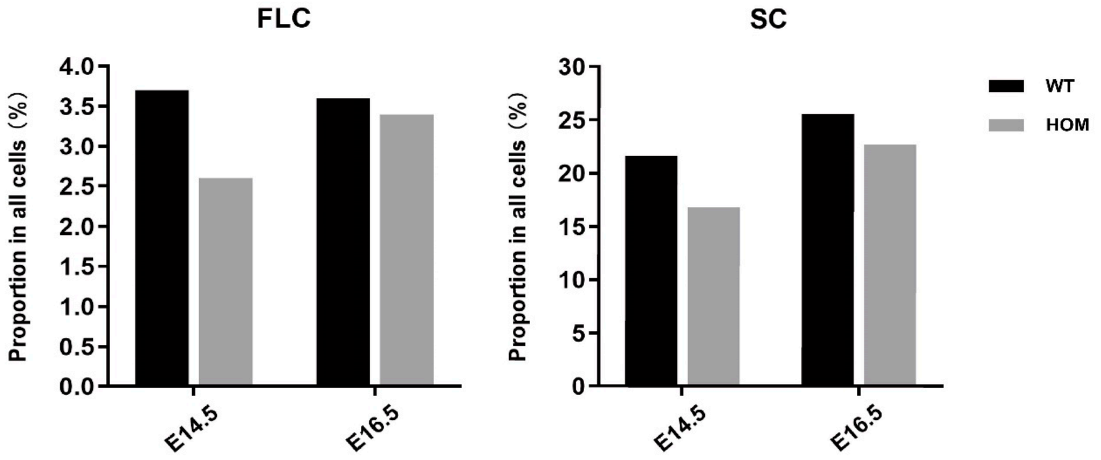

Figure S7. The proportion of FLC and SC in testicular cells at E14.5 and E16.5

The proportion of FLCs in testicular cells (left): at E14.5, the WT and HOM groups were 3.7% and 2.6%, respectively; at E16.5, the WT group was 3.6%, and the HOM group was 3.4%.

The proportion of SCs in testicular cells（right）: at E14.5, the WT and HOM groups were 21.6% and 16.8%, respectively; at E16.5, the WT group was 25.6%, and the HOM group was 22.7%.

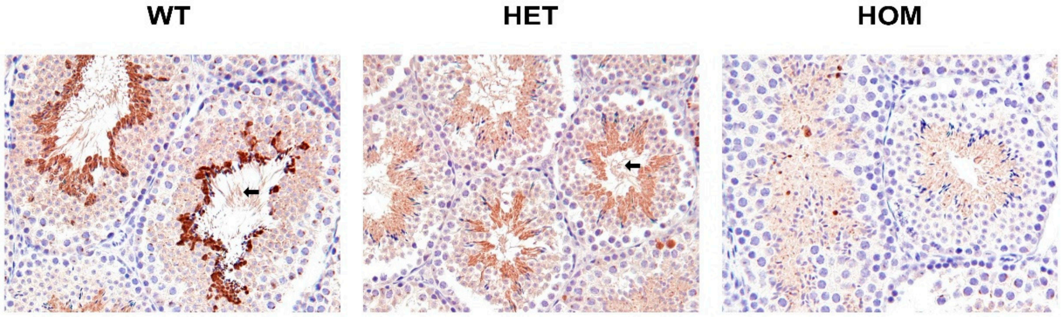

Figure S8. Immunohistochemistry of DNAH8 in mouse testis

DNAH8 (brown) was expressed in the center of seminiferous tubules, with particularly strong localization in the cytoplasm of round sperm cells and the flagella of elongated sperm cells in mouse testes. Compared to the WT group, the expression of DNAH8 was significantly reduced in the HET group. In the HOM group, no significant expression was detected. The black arrow indicates the flagella.

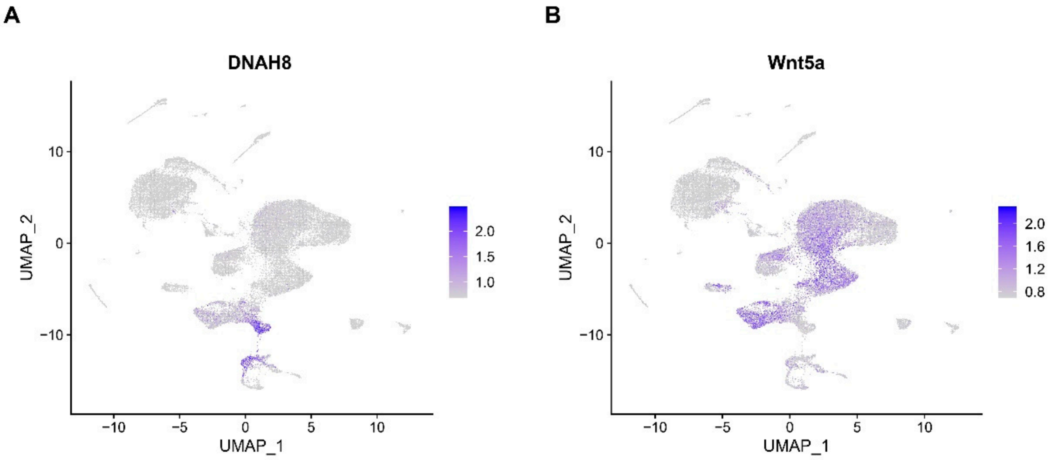

Figure S9. UMAP representations colored by expression levels of *DNAH8* and *Wnt5a* enriched in testis cell populations.

(A) UMAP plot showing the expression of *DNAH8*.

(B) UMAP plot showing the expression of *Wnt5a*.

A gradient of grey to blue indicates low to high expression levels.

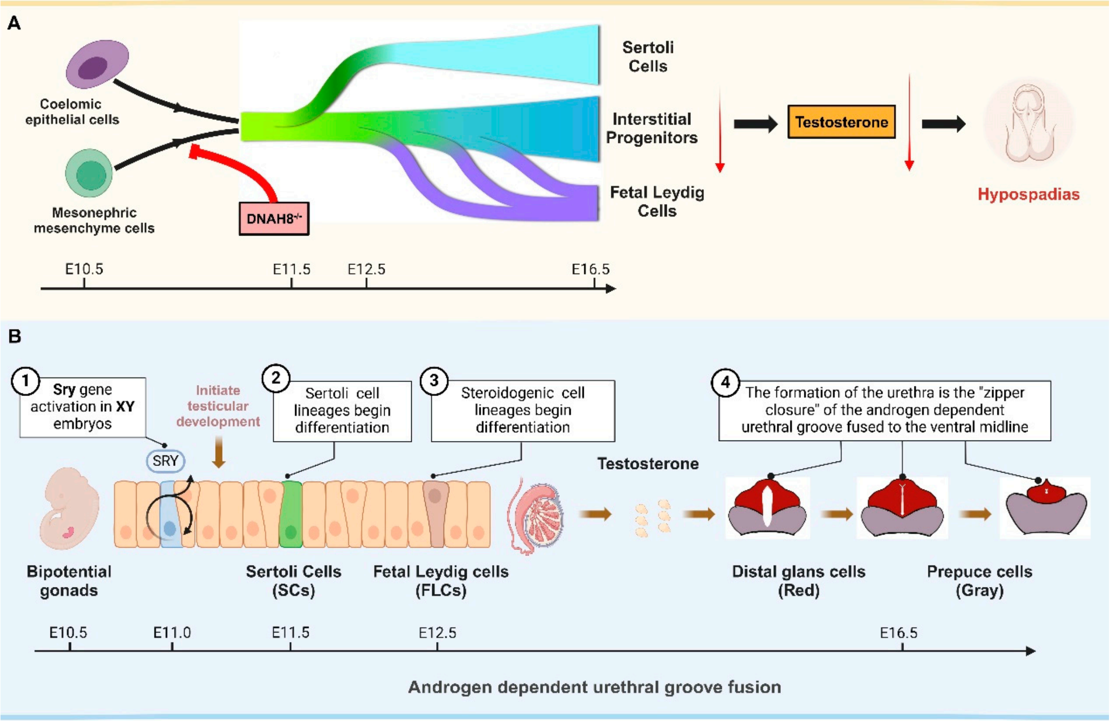

Figure S10. Potential mechanism of *DNAH8* in the development of hypospadias.

(A) The loss of DNAH8 function delayed the differentiation of the Sertoli cell lineage and steroidogenic cell lineage, resulting in a reduction in the number of SC and FLC and thus a decrease in testosterone synthesis and secretion. Low prenatal testosterone levels during the masculinization programming window led to abnormal urethral fusion by affecting androgen-dependent DDG and DVG cells.

(B) Androgen-dependent remodeling period of the normal development of the urethral formation.

Created in BioRender. Ding, Y. (2025) https://BioRender.com/v30u264.

Table S1. Primer sequences used for gene amplification in this study

Genotyping primer	NM-numbers	Primer Length (bp)	Melting temperature (°C)
DNAH8 P1 Forward CAGAGGTTTCTCCGTGGAGTC	NM_013811.4	21	57.80
DNAH8 P2 Reverse ATCCTGATGTCCCCGAGTGT	NM_013811.4	20	58.30
Dhh Forward ACCCCGACATAATCTTCAAGGA	NM_007857.5	22	55.38
Dhh Reverse GTTCACCCGCTCTTTGCAA	NM_007857.5	19	56.30
Ptch1 Forward CTAGCAATAGGGACCGCTCA	NM_001328514.1	20	56.73
Ptch1 Reverse GTCTCAGGGTAGCTCTCATAG	NM_001328514.1	21	53.85
Gli1 Forward TGTGGCGAATAGACAGAGGT	NM_010296.2	20	55.64
Gli1 Reverse TGCCAGATATGCTTCAGCCA	NM_010296.2	20	56.65
Gli2 Forward AGCCTTCACCCACCTTCTTG	NM_001081125.1	20	57.75
Gli2 Reverse TGGGCGCAGGCCCTCAGC	NM_001081125.1	18	68.68
Gli3 Forward CCGTTCAAAGCCCAGTACAT	NM_008130.3	20	55.24
Gli3 Reverse TGAGTAGGCTTTTGTGCAACC	NM_008130.3	21	55.59
Sf1 Forward TCTCTAACCGCACCATCAAG	NM_001403448.1	20	54.41
Sf1 Reverse TCGACAATGGAGATAAAGGTC	NM_001403448.1	21	51.33
Star Forward CCGGAGCAGAGTGGTGTCA	NM_011485.5	19	60.40
Star Reverse CAGTGGATGAAGCACCATGC	NM_011485.5	20	57.06
Cyp11a1 Forward CGCATAAAGCAGCAAAATTC	NM_001346787.1	20	59.25
Cyp11a1 Reverse	NM_001346787.1	20	58.84

ATGCGCTCCCCAAATATAAC			
Cyp17a1 Forward GATCTAAGAAGCGCTCAGGCA	NM_007809.3	21	57.26
Cyp17a1 Reverse GGGCACTGCATCACGATAAA	NM_007809.3	20	55.55
Ar Forward TAAAGACATTTTGAACGAGGCC	NM_013476.4	22	52.84
Ar Reverse GTCAGATATGGTTGAATTGCCC	NM_013476.4	22	53.68

Data S1 Gene expression list in each cell cluster of fetal external genitalia.

(CSV)

Data S2 The expression of specific marker genes in each cell subgroup of fetal external genitalia.

(CSV)

Data S3 The differential gene expression list of the DDG cell subgroup in the WT and HOM groups.

(CSV)

Data S4 The differential gene expression list of the DVG cell subgroup in the WT and HOM groups.

(CSV)

Data S5 The expression of specific marker genes in each cell subgroup of fetal testis.

(CSV)

Data S6 Label-free quantitative mass spectrometry on the testes of WT and HOM mice at E16.5.

(CSV)

KEY RESOURCES TABLE

REAGENT or RESOURCE	SOURCE	IDENTIFIER
**Antibodies**		
Anti-DNAH8 Antibody	Abcam	Cat# ab 121989
Anti-HSD3B1 Antibody	Abcam	Cat# ab 55268
Anti-Arx Antibody	Abcam	Cat# ab 308260
Anti-Postn Antibody	Abcam	Cat# ab 92460
Anti-Rspo3 Antibody	Invitrogen	Cat# PA5 - 100324
Anti-Ptgds Antibody	Invitrogen	Cat# PA5 - 95977
Anti-Wnt5a Antibody	Abcam	Cat# ab 229200
Anti-Rabbit IgG H&L (goat), Alexa Fluor 488	Abcam	Cat# ab 150077
**Chemicals, peptides, and recombinant proteins**		
4,6-diamidino-2-phenylindole (DAPI)	Servicebio	Cat# G1012
Animal Genomic DNA Quick Extraction Kit for PCR Analysis	Beyotime	Cat# D0065S
TaKaRa Ex Taq®	Takara	Cat# RR001A
Water-DEPC Treated Water	Sangon Biotech	Cat# B501005
4% Paraformaldehyde Fix Solution	Beyotime	Cat# P0099
1X PBS Buffer	Sangon Biotech	Cat# B540626
TSA scanning Kit	Servicebio	Cat# GP2035
**Critical commercial assays**		
Testosterone ELISA Kit	Beyotime	Cat# PT872
RNeasy Mini Kit	Qiagen	Cat# 74104
RNase-free DNase Kit	Qiagen	Cat# 79254
Prime Script™ RT Kit	Takara	Cat# RR037A

TB Green® Premix Ex Taq™ II Kit	Takara	Cat# RR820A
**Experimental models: Organisms/strains**		
Mouse:C57BL/6 DNAH8+/+	Shanghai Model Organisms Center	N/A
Mouse: C57BL/6 DNAH8-/+	Shanghai Model Organisms Center	This study
Mouse: C57BL/6 DNAH8-/-	Shanghai Model Organisms Center	This study
**Software and algorithms**		
Cell ranger v7.0.0	10X Genomics	https://www.10xgenomics.com
Prism 9	GraphPad	https://www.graphpad.com/
R 4.2.1	The R core Team	https://www.r-project.org/
SPSS V 21.0	IBM SPSS Inc.	https://www.ibm.com/spss

Supplementary materials and methods

Isolation of single testis and external genitalia cells

The testis and external genitalia tissues of E16.5 male fetal mice were surgically dissected, immersed in 10 ml of PBS, and shaken to remove residual blood cells, and enzymatic digestion was performed. Dead cells were counted by trypan blue (Sigma Aldrich T8154) on a Bio-Rad TC20 (Bio-Rad, California, USA), and samples with a viability of >85% were subjected to subsequent preparations.

Single-cell RNA-seq library preparation and sequencing

Single-cell RNA-seq libraries were prepared via a Chromium Single Cell 3' Kit v3 (PN-1000094, 10x Genomics, California, USA) according to the manufacturer's instructions. The final library was sequenced on the Illumina NovaSeq 6000 Sequencing System from LC-Bio Technology (Hangzhou, China). The default parameters in CellRanger software (v7.0.0, 10X Genomics, California, USA) were used for alignment analysis and gene expression information for each cell.

Quality control and sample integration

The Seurat package (v4.1.1) in R was used to create Seurat objects from the gene–cell matrix output by Cell Ranger. The cells were further filtered on the basis of the following threshold parameters: total number of expressed genes 200–7000, mitochondrial gene expression proportion <25%, and erythrocyte gene expression proportion <2.5%. Since our fetal rat testes and external genital tissues were obtained

and analyzed on the same batch, there is no need for further batch correction using the IntegrateData function in the Seurat package.

Cell identification and clustering analysis

To visualize the data, gene expression levels were calculated via the log normalization method in the "Normalization" function of Seurat software. Principal component analysis (PCA) was performed using the normalized expression levels and the top 20 PCs were also used for clustering and performing uniform manifold approximation and projection (UMAP) dimensionality reduction to distinguish cell subgroups. The FindClusters function in Seurat package with the resolution parameter set as 0.5 was used to cluster the cells. The differential expressed genes (DEGs) between the subgroups were identified via the Find Marker and Find All Markers functions with default parameters.

Differentially expressed gene calculation and gene enrichment analysis

The Seurat function FindAllMarkers (test.use = wilcox; min.pct = 0.1; logfc.threshold = 0.25) was used to identify differentially expressed genes (DEGs). Unless otherwise noted, the DEGs in each selected subcluster were calculated based on comparison between that subcluster and the rest of the dataset. The R package of the cluster profiler (version 4.2.2) and org.Mm.eg.db (pvalueCutoff = 0.05; qvalueCutoff = 0.2) were used to perform Gene Ontology (GO) analysis and Kyoto Encyclopedia of Genes and Genomes (KEGG) signaling pathway enrichment analysis on the DEGs.

Pseudotime analysis

The Slingshot package was used for pseudotime analysis. As mentioned earlier, PCA is used for dimensionality reduction, followed by UMAP visualization, and these reduced dimensions are used as inputs for pseudo time analysis. The slingshot() function to identify lineage trajectories and order the cells along the inferred paths. Specify the clustering labels for the starting and ending cells using the start.clus and end.clus parameters. Use the plot() function to plot trajectory maps to reveal potential trajectories of cells during development or state changes.

Ligand - receptor interaction

The Cell Chat package was used for ligand–receptor interaction analysis. The cell types were assigned based on the clustering results from the Seurat analysis, and a ligand-receptor database (CellChatDB) was used to map potential ligand-receptor pairs. To identify active ligand-receptor interactions, the CellChat object was processed with the CellChat function, which computes the communication networks among cell types by identifying potential ligand-receptor pairs and signaling pathways based on gene expression.
